# Pityriasis Versicolor

**Published:** 1901-07

**Authors:** 


### Pityriasis Versicolor.

---

Dr. Jacob Sobel calls attention to the frequency with which pityriasis versicolor is overlooked, in spite of the fact that a diagnosis may be made with ease by the use of Allen's test (the application of Lugol's solution, which gives a black or deep mahogony color on the diseased surface). He believes the old idea that the disease attacks only covered surfaces is a mistaken one, and that phthisical subjects seem to be attacked more often than others merely because the frequency with which their chests are exposed during examination furnishes a better opportunity for making a diagnosis.—*Philadelphia Medical Journal.*

